# Human leukocyte antigen class II gene diversity tunes antibody repertoires to common pathogens

**DOI:** 10.3389/fimmu.2022.856497

**Published:** 2022-08-08

**Authors:** Taushif Khan, Mahbuba Rahman, Ikhlak Ahmed, Fatima Al Ali, Puthen Veettil Jithesh, Nico Marr

**Affiliations:** ^1^ Research Branch, Sidra Medicine, Doha, Qatar; ^2^ College of Health and Life Sciences, Hamad Bin Khalifa University, Doha, Qatar

**Keywords:** human antibody repertoires, microbial infection, phage immunoprecipitation sequencing, human leukocyte antigen, major histocompatibility complex, polymorphisms, allelic diversity, association study

## Abstract

Allelic diversity of human leukocyte antigen (HLA) class II genes may help maintain humoral immunity against infectious diseases. In this study, we investigated germline genetic variation in classical HLA class II genes and employed a systematic, unbiased approach to explore the relative contribution of this genetic variation in the antibody repertoire to various common pathogens. We leveraged a well-defined cohort of 800 adults representing the general Arab population in which genetic material is shared because of the high frequency of consanguineous unions. By applying a high-throughput method for large-scale antibody profiling to this well-defined cohort, we were able to dissect the overall effect of zygosity for classical HLA class II genes, as well as the effects associated with specific HLA class II alleles, haplotypes and genotypes, on the antimicrobial antibody repertoire breadth and antibody specificity with unprecedented resolution. Our population genetic studies revealed that zygosity of the classical HLA class II genes is a strong predictor of antibody responses to common human pathogens, suggesting that classical HLA class II gene heterozygosity confers a selective advantage. Moreover, we demonstrated that multiple HLA class II alleles can have additive effects on the antibody repertoire to common pathogens. We also identified associations of HLA-DRB1 genotypes with specific antigens. Our findings suggest that HLA class II gene polymorphisms confer specific humoral immunity against common pathogens, which may have contributed to the genetic diversity of HLA class II loci during hominine evolution.

## Introduction

Originally discovered as the genetic loci responsible for rapid graft rejection, the classical major histocompatibility complex class I (*MHC-I*) and class II (*MHC-II*) genes encode glycoproteins responsible for antigen presentation, allowing the immune systems of all jawed vertebrates to discriminate between self and non-self molecules. In humans, the classical *MHC* genes are located with functionally related genes on chromosome region 6p21.3; this cluster of genes is referred to as the human leukocyte antigen (HLA) gene complex (1). The classical HLA class I glycoproteins are ubiquitously expressed and contain the functional sites that primarily bind endogenous peptides, thereby contributing to innate immunity by engaging natural killer cell receptors, and to adaptive cellular immunity, through the engagement of the αβ antigen receptors on cytotoxic (CD8^+^) T cells. In contrast, the classical HLA class II glycoproteins, HLA-DR, -DP and -DQ, are primarily expressed by antigen presenting cells (with some exceptions, such as tumor cells). These molecules contribute to adaptive immunity by presenting exogenous peptides and engage with the αβ antigen receptors of helper (CD4^+^) T cells, which in turn participate in the activation of naïve B cells (1). Thus, the HLA class II glycoproteins play an indirect but critical role in antibody responses to thymus-dependent antigens. Normally, the peptides presented by the HLA class I and II glycoproteins are derived from host proteins that do not elicit any immune responses due to the elimination of self-reactive T cells during their development in the thymus. This process is orchestrated by the interaction of immature T cells with a variety of thymic cell types. However, following infection or in cancer cells, the binding of non-self (pathogen or mutated) peptides by the HLA glycoproteins leads to activation of naïve or memory T cells (2, 3).

In comparison to most other human genes, the classical HLA loci are extremely polymorphic as a consequence of pathogen-driven balancing selection pressure over prolonged time periods. Some of these polymorphisms were shown to precede the speciation of modern humans (i.e., trans-species polymorphisms), or were introduced into the human gene pool by admixture between archaic and modern humans (i.e., adaptive introgression) (1, 4–8). To date, more than 25,000 HLA allele sequences have been identified (9). Variation is highest at sites that define the peptide-binding repertoire (5). Multiple selection mechanisms have been proposed to underly this extraordinarily high level of genetic diversity of classical HLA loci, including negative frequency-dependent selection (also referred to as rare allele advantage), heterozygote advantage, and fluctuating selection, none of which are mutually exclusive (1, 5). Nevertheless, providing empirical evidence for the underlying selection mechanisms through human studies and evaluating their relative contribution to HLA diversity has not been straightforward (5). Similarly, pinpointing causal variant-disease relationships (or causal variant-phenotype relationships) remains a challenge due to the additive effects of multiple HLA loci that have related functions, with each of the classical HLA loci on its own exhibiting a high degree of immunological redundancy, as well as due to the density and strong linkage disequilibrium (LD) of HLA genes (1, 4, 5, 10).

The functional effects of common polymorphisms in HLA loci or elsewhere in the human genome have mainly been inferred using an epidemiological study design, in which a group of selected cases with a study-defined disease or individuals with a specific immunological phenotype (e.g., a vaccine response or lack thereof) are compared to a group of controls to identify those polymorphisms and alleles that are statistically over-represented among either the case group (i.e., risk alleles) or the controls (i.e., protective alleles). Such studies have revealed associations of certain HLA class I gene polymorphisms with human immunodeficiency virus-type 1 (HIV-1) virus load and AIDS progression (11, 12). Associations have also been identified between HLA class II gene variants and chronic hepatitis B and C virus (HBV and HCV) infections, leprosy and tuberculosis, or responses to influenza and hepatitis B vaccination, albeit most identified risk or protective alleles have only small-to-modest effect sizes [reviewed in (13)]. Moreover, specific HLA alleles have been associated with a variety of autoimmune and inflammatory diseases (13). These associations highlight the delicate balance between the ability of the immune system to activate potent effector mechanisms against invading pathogens while preventing excessive host tissue damage (14).

Nevertheless, our current understanding of the inter-individual variation of the immune responses to microbial challenges remains limited. The relative contributions of different genetic and non-genetic factors driving this variation are only beginning to be unraveled using holistic (i.e., systems immunology) approaches applied to larger cohorts of either healthy individuals, or the general population of a given geographic region (or ethnicity). These approaches allow the dissection of the gene-phenotype relationships underlying the enormous inter-individual differences in susceptibility to pathogens at a much higher resolution (15). To date, only a few studies have investigated the functional consequences of genetic variation in HLA class II genes on the variability of antibody responses in healthy individuals or the general population (16–18). Such studies have been hampered not only by the large number of different HLA class II alleles, the strong LD and the high immunological redundancy of individual HLA class II genes, but also the lack of cost-effective and technically feasible experimental approaches that enable the assessment of very large numbers of antibody-antigen interactions in sufficiently sized human cohorts.

In this study, we explored the relative contribution of specific HLA class II alleles, haplotypes and genotypes on the variation of human antibody responses to a variety of common human pathogens. We conducted an unbiased, large-scale, high-throughput screen of antigen-antibody interactions using phage immunoprecipitation sequencing (PhIP-Seq) (19, 20) and samples from a well-defined cohort of 800 adult Qatari nationals and long-term residents of Qatar. This sample of the general population was expected to have limited genetic diversity and an excess of individuals with HLA homozygosity due to high rates of consanguinity (21–24), thereby allowing us to overcome challenges related to the extreme allelic diversity of classical HLA class II loci.

## Materials and methods

### Study cohort

The study cohort of 800 adult male and female Qatari nationals and long-term residents of Qatar was randomly selected from a larger cohort of individuals taking part in a longitudinal study of the Qatar Biobank (QBB) (21) as described previously (25). Relevant demographic data of the study subjects have been described previously (25).

### HLA type inference from whole genome sequencing data

Whole genome sequencing (WGS) of our study cohort was performed as part of the Qatar Genome Programme (QGP) (https://qatargenome.org.qa/), with a minimum average coverage of 30× (26). Sequencing read data were generated and processed as described elsewhere (27). In brief, sequencing libraries were generated from whole blood-derived fragmented DNA using the TruSeq DNA Nano kit (Illumina, San Diego, CA, USA) and sequence reads were generated using a HiSeq X Ten1 system (Illumina). Primary sequencing data were demultiplexed using bcl2fastq (Illumina) and quality control of the raw data was performed using FastQC [v0.11.2] (Babraham Bioinformatics, Babraham Institute, Cambridge, UK). Sequence reads were aligned to the human reference genome sequence [build GRCh38] using Sentieon Genomics pipeline tools (Sentieon, San José, CA, USA). HLA type inference for association studies was performed using two independent typing methods with high accuracy on the WGS data, namely HLA*LA (28) and HLA-HD (v 1.4.0) (29). For both methods, we resolved HLA alleles at 2-field resolution (corresponding to the protein sequence) using an updated HLA allele dictionary from the IPD-IMGT/HLA database (v. 3.45) and default options as described in the respective repositories (30, 31) (**Supplementary Table 1**). To account for typing errors and to ensure sufficient statistical power for our downstream association studies, we excluded alleles with a minor allele frequency (MAF) <0.01 as well as alleles for which the difference in the allele frequencies determined by HLA*LA and HLA-HD at 2-field resolution was ≥5% (**Supplementary Figure 1**).

### Linkage analysis, heterozygosity rates, population differentiation, and homozygosity estimation

LD of classical HLA loci was quantified using eLD (32). Individual heterozygosity rates were calculated using PLINK [version 1.9] (33). More specifically, we used the –het command to count the observed (O_HOM_) and expected (E_HOM_) autosomal homozygous sites for SNVs with a MAF >5% in each individual. The individual heterozygosity rate was defined as (E_HOM_ – O_HOM_)/E_HOM_, as described previously (34). The expected number of homozygotes for a given HLA class II allele was estimated based on the imputed allele frequencies and assuming Hardy–Weinberg equilibrium. Deviation from the Hardy–Weinberg equilibrium was assessed using Fisher’s exact test and the Bonferroni method was used to correct for multiple testing. A -log_10_(*P*-value) ≥4.7 was considered to indicate statistical significance.

### Phage immunoprecipitation sequencing (PhIP-Seq) and peptide enrichment analysis

The VirScan phage library used for PhIP-Seq in the present study was obtained from S. Elledge (Brigham and Women’s Hospital and Harvard University Medical School, Boston, MA, USA). PhIP-Seq of serum samples from the 800 study subjects and peptide enrichment analysis were performed as described previously (19, 20, 25). In brief, we utilized an expanded version (35) of the original VirScan phage library described by Xu et al. (20). Custom sequencing libraries were prepared as previously described (19) and sequencing was performed using a NextSeq system (Illumina). To filter for significantly enriched peptides, we imputed -log_10_(*P*-values) by fitting a zero-inflated generalized Poisson model to the distribution of output counts and regressed the parameters for each peptide sequence based on the input read count. Peptides with a reproducibility threshold exceeding 2.3 [-log_10_(*P*-value)] for two technical sample replicates were considered significantly enriched. We then computed microbial score values as described by Xu et al. (20) by counting the number of non-homologous, significantly enriched peptides per species (i.e., we counted enriched peptides per microbial species that did not share linear sequence identity in seven or more amino acids, the estimated size of a linear B cell epitope). The scores were finally adjusted by dividing them according to previously established species-specific significance cut-off values, to account for the varying number of peptides and potential protein antigens for each microbial species encompassed by the phage display library (25). As such, the adjusted species score values served as a measure of the breadth of the antibody repertoire (i.e., reflecting the diversity of antibodies) against a pathogen. Samples with an adjusted species score ≥1 were considered seropositive for the corresponding microbial species. The seroprevalence was calculated for each species as the number of seropositive samples divided by the total number of samples in the cohort. Similarly, we estimated seroprevalence values for each sex. In our downstream analysis, we excluded antibody specificities to species for which the seroprevalence in the local adult population was <5%.

### Association studies

We examined the contribution of the genetic variation in the classical HLA class II loci to the diversity of the antibody repertoire at different resolutions (i.e., by independently assessing the effect of zygosity, haplotypes, alleles and HLA-DRB1 genotypes). The adjusted species scores served as a measure of the breadth of the antibody repertoire against each of the 48 microbial species evaluated in this study, and generalized linear models (GLMs) or logistic linear regression models were applied (see below). We adjusted *P*-values to account for multiple testing using the Holm method (36). Coefficients of association (β) were reported using a natural log scale. Stringent thresholds were chosen to filter for statistically and biologically meaningful associations, considering both magnitude of association (β) and significance (*P*-value). A |β| ≥0.68 (i.e., the natural log-transformed representation of an odds ratio of >2 or <0.5) and an adjusted *P*-value ≤0.005 (indicating 0.5% chance of type 1 error) was considered to indicate statistical significance. The combination of stringent thresholds for β and the adjusted *P*-value increases the probability of identifying associations with biological relevance.

For each association model, we used a combined method for over- and under-sampling to mitigate the effect of high-level binary class imbalance, as previously described (37). In brief, we first performed over-sampling of the minor class using the Synthetic Minority Over-sampling Technique (SMOTE), which generated new synthetic minority instances by interpolating close-by pre-existing positive samples. We then performed under-sampling to reduce the noise from over-sampling events by using the Edited Nearest Neighbors (ENN) method. The combination of SMOTE and ENN has been shown to effectively reduce the effects of class imbalance in highly imbalanced datasets (38, 39). Finally, we performed a cross-validation with 100-fold bootstrapping for each association model. After extracting significant associations from each test based on the criteria detailed above, significant associations which were consistently found in more than 50% of the simulated sample iterations were retained, and parameters of associations were aggregated as median values.

We also defined the anti-microbial response ratio (RR) as a new feature for the assessment of HLA class II alleles, haplotypes, or HLA-DRB1 genotype groups. The RR for a given HLA class II allele was calculated by dividing the number of significant associations of the allele examined (i.e., when significance was found using both HLA typing methods and taking into account the direction of association) by the total number of microbial species for which we identified at least one significant association with any HLA class II alleles assessed in this study. The RRs for haplotypes or HLA-DRB1 genotype groups were calculated similarly. Accordingly, the anti-microbial RR allowed us to assess associations between specific features (i.e., HLA class II alleles, haplotypes, or genotypes) and the antibody responses against multiple microbial species. It also takes into consideration that a given feature might be positively associated with the antibody response against one (or more than one) species and at the same time, negatively associated with the antibody response of another species. We applied a threshold of |0.3| to further distinguish biologically relevant and significant associations from marginal associations.

#### Associations with non-genetic features

To test for associations between the antibody repertoire breadth, age and sex, we used a GLM according to the following equation:


$$\text{Adjusted species score}=\beta_{1}*variable+\;\beta_{age}*Age_{norm}+\;\beta_{sex}*\left[sex\right]$$

#### Associations with zygosity

To assess the effect of zygosity of classical HLA class II genes on the breadth of the antibody repertoire against common pathogens, we performed a logistic linear regression using the following equation:


$$\text{Zygosity}=\;\beta_{1}*variable+\;\sum_{i}^{species}\beta_{i\;}*\;adjusted\;species\;score_{i}+\;\beta_{3}*covariates+\epsilon_{1}$$

We treated the zygosity state in the HLA class II genes *HLA-DPA1*, *-DPB1*, *-DQA1*, *-DQB1*, and *-DRB1* as binary dependent variables (i.e., “0” if an individual was homozygous for a given allele, or “1” if heterozygous). As such, a positive association indicated a directional relationship between an increased antibody repertoire breadth and a heterozygous genotype. The adjusted species scores were used as independent variables. Age, sex and the first four genetic principal components (to account for the population sub-structure, which may lead to spurious associations) were used as covariates (**Supplementary Figure 2** and **Supplementary Table 2**). The genetic principal components were calculated as described elsewhere (34) considering all common variants in our cohort (genome-wide) with an MAF ≥0.05. We choose to include the first four principal components as covariates in our association models which, when combined, reflected 52.9% of the germline-genetic variance in our cohort. Each additional genetic principal component contributed less than 10% of genetic variance (not shown) and was therefore not considered as covariate to limit unnecessary complexity of our models.

#### Associations with HLA class II alleles, haplotypes and HLA-DRB1 genotypes

To test for associations between the antibody repertoire breadth and HLA class II alleles, HLA-DQA1~DQB1~DRB1 haplotypes, and HLA-DRB1 genotypes, we used GLMs according to the following equations:


$$\text{Adjusted species score}=\;\beta_{1}*variable+\sum_{i}^{haplotype}\beta_{i\;}*haplotype_{i}+\;\beta_{3}*covariates+\epsilon_{1}$$

$$\text{Adjusted species score}=\;\beta_{1}*variable+\sum_{i}^{allele}\beta_{i\;}*\;allele_{i}+\;\beta_{3}*covariates+\epsilon_{1}$$

$$\text{Adjusted species score}=\;\beta_{1}*variable+\;\sum_{i}^{genotype}\beta_{i\;}*\;genotype_{i}+\;\beta_{3}*covariates+\epsilon_{1}$$

Only HLA class II alleles and HLA-DRB1~DQA1~DQB1 haplotypes with a frequency ≥1% were assessed. Age, sex and the first four genetic principal components were used as covariates. To further mitigate the potential effects of typing errors (e.g., at the sample level), we tested for associations assigned by HLA*LA and HLA-HD independently and then prioritized the associations identified by both methods.

### Differential enrichment analysis of antibody-antigen interactions across DRB1 genotypes

To examine the differential enrichment at the peptide and antigen level, we first performed pairwise differential enrichment tests per peptide, accounting for all possible pairwise comparisons of the DRB1 genotype groups identified (n = 15). We considered only peptides that were significantly enriched in at least two samples among the total number of samples tested. Accordingly, for each peptide assessed, we performed 120 pairwise differential enrichment tests based on the equation (n×(n-1))/2. Using these filter criteria, we tested a total of 4,303 enriched peptides when considering all the DRB1 genotype groups combined. Next, we screened for differential enrichment of antibody-antigen interactions in each tested DRB1 group-pair using an |OR| ≥2 and a *P*-value ≤0.005 (Fisher’s exact test) as the cut-off. After removing peptides from microbial species with a seroprevalence <5%, 224 differentially enriched peptides (DEPs) were included in our downstream analysis. We then assessed the variance of significant antibody-antigen interactions (i.e., per UniProtKB entry) across DRB1 genotype groups. To do so, we first estimated the peptide enrichment frequency of each DEP per DRB1 genotype group. This peptide enrichment frequency was calculated as the ratio of the number of samples in the DRB1 genotype group for which a DEP was significantly enriched, divided by the total number of samples in that group. Next, we calculated the mean of the peptide enrichment frequency per UniProtKB entry for each DRB1 group. Finally, we assessed the variance in this mean value for each Uniprot entry and DRB1 group to identify the antibody-antigen interactions with the highest variance across different DRB1 groups. For this purpose, we only considered UniProtKB entries for which the variance distribution was above the 75^th^ quartile and at least two DEPs were identified. Finally, we filtered for UniProtKB entries for which DEPs were less frequent (<5%) among individuals in at least one of the DRB1 genotype groups.

## Results

### HLA type inference from whole genome sequencing data of the 800 study participants

We estimated the allelic state of the classical HLA class II genes in our study cohort of 800 Qatari nationals and long-term residents of Qatar from WGS data using two independent HLA typing methods, namely HLA*LA and HLA-HD. **Table 1** shows the demographic information of the study subjects. To mitigate spurious associations due to possible errors in typing HLA alleles inferred from WGS data (40, 41) and to ensure sufficient statistical power for our downstream association studies, we considered only common alleles with a minor allele frequency (MAF) ≥1% and highly concordant typing results obtained using the two typing methods at 2-field resolution (for details, see Materials and Methods, and **Supplementary Figure 1**). As expected, *HLA-DRB1* was the most polymorphic gene among the HLA class II genes, followed by *HLA-DQB1*, *HLA-DPB1* and *HLA-DQA1* (**Supplementary Figure 1D**). The *DRB1* alleles most commonly present (≥10%) were HLA-DRB1*03:01 (15.93%), HLA-DRB1*07:01 (15.06%) and HLA-DRB1*16:02 (10.50%) (**Supplementary Table 3**). A multiple sequence alignment of the gene products of all HLA-DRB1 alleles analyzed in this study is shown in **Supplementary Figure 3**. As expected, we detected strong LD among the genetic variants in the class II loci (**Supplementary Figure 4**), demonstrating that the HLA class II alleles are strongly associated in the population and are inherited as haplotypes (**Supplementary Table 4**). None of genotype frequencies assessed in this study deviated from the Hardy–Weinberg equilibrium (not shown).

**Table 1:** Demographic information of the study subjects.

Nationality	Gender	Number of subjects	Age (Years)		
			Mean	Min	Max
Qatari	Female	501	41	19	81
Qatari	Male	282	41	19	81
Bahrain	Female	2	36	35	36
Bahrain	Male	1	57	NA	NA
Syria	Male	3	44	42	46
Yemen	Female	3	31	28	33
Yemen	Male	1	23	NA	NA
Saudi Arabia	Female	3	39	19	60
Jordan	Female	1	40	NA	NA
Jordan	Male	1	43	NA	NA
Morocco	Female	1	46	NA	NA
Kuwait	Female	1	24	NA	NA

NA, not applicable.

### Characterization of antibody responses to common human pathogens

Next, we performed PhIP-Seq (19, 20) on serum samples obtained from each individual (n = 800) of our study cohort at a single time-point [i.e., at the time of recruitment to the QBB study (21)]. In brief, this technology enabled us to obtain a comprehensive profile of antibody repertoires in our study cohort using phage display of oligonucleotide-encoded peptides, followed by immunoprecipitation and massive parallel sequencing (19, 20). The VirScan phage library used for PhIP-Seq in the present study comprised tiles of peptides up to 56 amino acids in length that overlap by 28 amino acids and collectively encompass the full proteomes of most known human-tropic viruses (approximately 400 species) plus many bacterial protein antigens (35). Using this technique, we identified the antibody repertoires of 798 individuals [data from two individuals were excluded from the downstream analysis as these did not meet our stringent criteria for quality control (25)]. We also excluded antibody specificities against species for which the seroprevalence in the local adult population was less than 5% (for details see the Materials and Methods section). We retained antibody specificities against a total of 48 microbial species for our downstream analysis (**Table 2**). As expected, the majority of individuals were seropositive for antibodies against various human-tropic viruses that frequently cause upper respiratory tract infections (i.e., ‘common cold’ viruses), and human herpesvirus (HHV) species, which commonly establish life-long persistent infections (i.e., latency), as well as bacteria such as *Staphylococcus aureus, Streptococcus pneumoniae*, and *Mycoplasma pneumoniae*, which frequently colonize the skin or upper airways but are typically innocuous. We also detected antibodies against human papillomaviruses (HPVs), which cause common warts, enteroviruses (EV) (i.e., EV-A, -B and -C), rotavirus A and *Helicobacter pylori*, which can cause gastrointestinal disease, as well as antibody responses that are likely to reflect immunity from childhood vaccination (e.g., to smallpox and polio vaccine strains) (**Table 2**).

**Table 2:** Frequently detected antimicrobial antibody responses.

Species	Overall (%)	Female (%)	Male (%)
*Streptococcus pneumoniae*	95.9	96.1	95.5
Rhinovirus B	93.7	93.5	94.1
Human herpesvirus 4	93.0	93.5	92.0
*Staphylococcus aureus*	92.9	93.7	91.3
Human herpesvirus 5	90.2	92.6	86.1
Human herpesvirus 1	74.1	76.1	70.4
Rhinovirus A	73.8	70.6	79.4
Human respiratory syncytial virus	68.4	68.7	67.9
Human adenovirus C	56.6	59.3	51.9
*Mycoplasma pneumoniae*	53.8	53.0	55.1
Human herpesvirus 6B	47.6	53.0	38.0
Human parainfluenza virus 3	44.4	44.8	43.6
Human herpesvirus 3	43.0	41.5	45.6
Human herpesvirus 7	43.0	44.2	40.8
Human herpesvirus 2	40.2	39.7	41.1
Enterovirus B	38.7	37.8	40.4
Human herpesvirus 8	38.6	39.5	36.9
Influenza A virus	37.3	35.2	41.1
Enterovirus A	35.3	33.3	39.0
Human metapneumovirus	34.7	34.2	35.5
Enterovirus C	30.2	29.4	31.7
Influenza B virus	29.9	26.0	36.9
Vaccinia virus	28.4	28.4	28.6
Human coronavirus HKU1	25.9	25.8	26.1
Norwalk virus	25.6	29.2	19.2
Human herpesvirus 6A	24.2	25.0	22.6
Human adenovirus F	24.2	22.9	26.5
Human adenovirus D	23.9	23.7	24.4
*Helicobacter pylori*	19.5	20.5	17.8
Cosavirus A	15.2	15.9	13.9
Influenza C virus	14.5	15.5	12.9
Hepatitis B virus	14.4	14.3	14.6
Rotavirus A	14.4	14.1	15.0
Alphapapillomavirus 10	14.4	17.0	9.8
Cowpox virus	14.2	15.1	12.5
Adeno-associated dependoparvovirus A	12.8	11.4	15.3
Human adenovirus B	12.7	11.9	13.9
Human parvovirus B19	12.7	12.9	12.2
Alphapapillomavirus 9	12.5	12.5	12.5
Sapporo virus	12.3	11.5	13.6
Human parainfluenza virus 1	10.8	11.5	9.4
Aichivirus A	10.3	9.0	12.5
Human coronavirus NL63	8.9	9.2	8.4
Human parainfluenza virus 2	7.8	8.2	7.0
Human adenovirus E	7.6	6.7	9.4
Alphapapillomavirus 6	7.5	8.0	6.6
Human adenovirus A	6.9	6.3	8.0
Human coronavirus 229E	5.6	4.3	8.0

### Impact of age and sex on the species-specific antibody responses

Previous studies of the French *Milieu Interieur* cohort showed that age and sex are important non-genetic covariates underlying the inter-individual variability of human antibody responses to common pathogens among healthy individuals (18). To assess the effect of age and sex on the antibody repertoires against common pathogens among the individuals in our cohort, we applied a GLM. To account for the varying number of peptides and potential protein antigens for each microbial species encompassed by the phage display library, we adjusted the species-specific scores by normalizing the counts of significantly enriched, non-homologous peptides (i.e., pulled down peptides containing distinct linear B cell epitopes) against the total count of peptides for a given microbial species represented in the phage library, as described previously (25) (**Supplementary Table 5**). We found that the breadth of the antibody repertoire against HHV-1, -2, -4 and -5 was significantly and positively associated with age, whereas the antibody repertoire breadth against human rhinoviruses (HRV)-A and -B, EV-A, human adenovirus (HAdV)-C, HHV-6B and *S. pneumoniae* correlated negatively with increasing age. We also found a marginal but measurable sex-specific bias suggesting a slight increase in the antibody repertoire breadth against influenza B virus (IBV) and HRV-A among males, whereas the oppositive was the case for the antibody repertoire breadth against HHV-4, *H. pylori* and *S. pneumoniae* (**Supplementary Table 6**). We therefore included age and sex along with genetic principal components as covariates in all subsequent genetic association studies.

### Zygosity of classical HLA class II genes affects the antimicrobial antibody repertoire breadth

**Figure 1:**
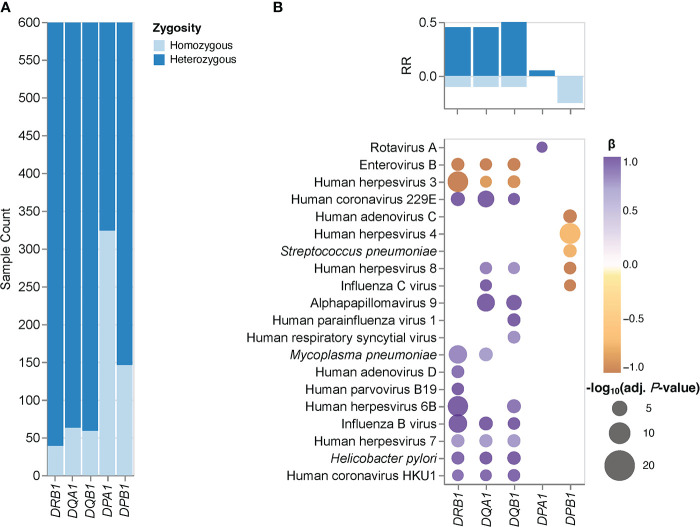
HLA class II gene zygosity effect on the antibody repertoire breadth against common microbial infections. **(A)** Bar plots depict the fraction of homozygous (light blue) and heterozygous (dark blue) individuals (n = 599) for each HLA class II gene tested. **(B)** Heatmaps depicting significant associations (adjusted *P*-value ≤ 0.005) between zygosity in HLA class II genes and the antibody repertoire breadth against common microbial infections. The coefficient (β) and direction of associations are indicated by a color gradient for each symbol (a positive β value indicates a positive association with a heterozygous genotype and visa versa). Bar plots depict the anti-microbial response ratio (RR) associated with a heterozygous genotype (positive values) and homozygous genotype (negative values) for each gene tested.

Next, we assessed associations between zygosity of classical HLA class II genes and the antimicrobial antibody repertoire breadth in our cohort. To mitigate the potential impact of HLA typing errors, we first selected those individuals for which the *HLA-DRB1*, *-DQA1*, *-DQB1*, *-DPB1*, and *-DPA1* genotypes inferred by HLA*LA and HLA-HD were concordant; a total of 599 individuals met this criterion. This sub-cohort also included a small, but sufficiently sized fraction of individuals who were homozygous for the highly polymorphic *HLA-DRB1* gene, and as expected, larger fractions of individuals who were homozygous for the less polymorphic *HLA-DQB1*, *- DQA1*, *-DPB1* and *-DPA1* genes (**Figure 1A**). HLA diversity among these subjects was more limited at the individual level compared to that of the HLA heterozygotes (**Supplementary Table 1**). Consequently, these individuals express fewer molecular variants of the HLA-DP, -DQ, and -DR heterodimers that present peptides to CD4^+^ T cells. We therefore hypothesized that HLA class II gene homozygotes, particularly those individuals who are homozygous for the highly polymorphic *HLA-DRB1* gene, may also have a lower capacity for generating antibody responses against a broad spectrum of antigens than their heterozygous peers, at least in response to some pathogens. Then, we independently assessed the effects of *HLA-DRB1*, *-DQA1*, *-DQB1*, *-DPB1*, or *-DPA1* zygosity on the antibody repertoire breadth against each of the 48 common microbial species listed in **Table 2** by performing logistic regression analyses using HLA class II gene zygosity as response variables, the adjusted species-specific scores (measure of antibody repertoire breadth) as explanatory variables, and age, sex and the first four genetic principal components as covariates. We also controlled for the potential effects of imbalanced sample sizes (see Materials and Methods for details). After applying stringent statistical criteria and controlling for multiple testing, we identified 40 associations between the zygosity state of *HLA-DRB1, -DQA1*, *-DQB1*, *-DPA1* or *-DPB1*, and the antibody repertoire breadth against 20 microbial species (**Figure 1B**). Notably, heterozygosity for the highly polymorphic genes *HLA-DRB1, -DQA1* and *-DQB1* was strongly and positively associated with the antibody repertoire breadth against a variety of common human viruses, including HHV-6B, HHV-7, HHV-8, alphapapillomavirus 9, IBV, ICV, endemic human coronaviruses (HCoV)-229E and -HKU1, and bacterial pathogens, such as *H. pylori* and *M. pneumoniae.* Interestingly, we also identified a negative association between heterozygosity in *HLA-DPB1* and the antibody responses against HHV-4, HHV-8, ICV, HAdV-C and *S. pneumoniae*, as well as between heterozygosity in *HLA-DQB1*, *-DQA1* and *-DRB1* and the antibody repertoire breadth specifically to HHV-3 and EV-B (**Figure 1B**).

### HLA class II allele- and HLA-DRB1~DQA1~DQB1 haplotype-specific effects on the antibody repertoire breadth against common microbial infections

**Figure 2:**
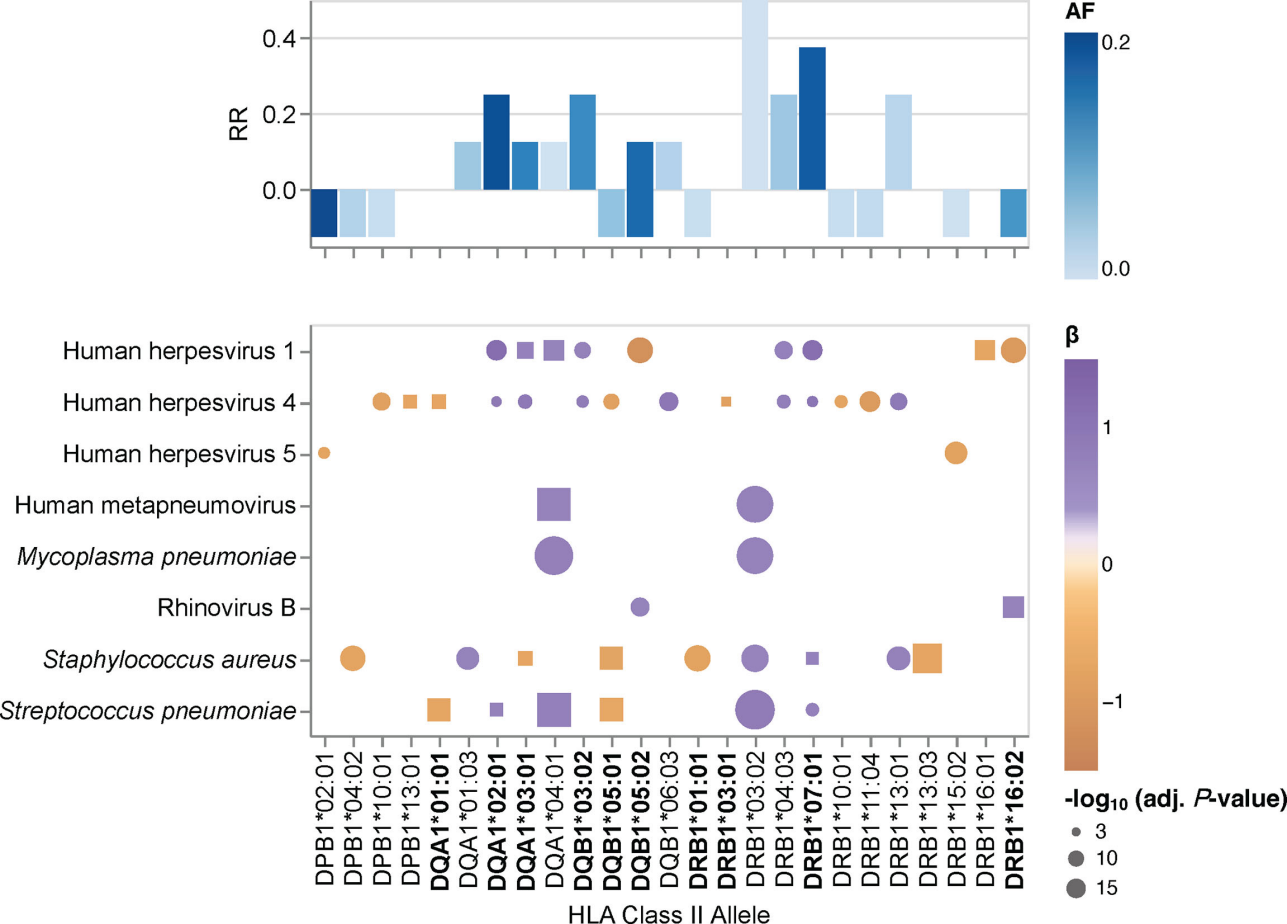
HLA class II allele-specific effects on the antibody repertoire breadth against common microbial infections. Heatmaps depicting significant associations (adjusted *P*-value ≤0.005) between specific alleles and the antibody repertoire breadth against common microbial infections. The coefficient (β) and direction of associations are indicated by a color gradient for each circle. The symbol size depicts the -log_10_(adjusted *P*-value) of the association. Round symbols show aggregated *P*-values for associations that reached statistical significance when using results from both HLA typing methods (i.e., HLA*LA and HLA-HD) as explanatory variables. Square symbols show *P*-values when associations reached statistical significance with only one of the two HLA typing methods used (i.e., either HLA*LA or HLA-HD). Associations for alleles labeled in bold were also found for the corresponding haplotypes listed in **Supplementary Table 7**. Bar plots depicting the antimicrobial response ratio (RR) for each allele or haplotype. The allele frequency is indicated by a color gradient for each bar.

Given the pathogen-driven balancing selection and allelic diversity of classical HLA class II loci, particularly at sites that define the peptide-binding repertoire (5), we speculated that classical HLA class II alleles and haplotypes have varying effects on the breadth of antibody repertoires detected in our study cohort, which may also differ depending on the microbial species. These microbial species, for which the variance in human antibody responses between individuals with different HLA class II alleles and haplotypes is greatest, may arguably also play an important role in driving allelic diversity of HLA class II genes in the first place. We tested for associations between specific HLA class II alleles and the antibody repertoire breadth against the 48 common microbial species listed in **Table 2** by using the adjusted species scores as response variables and the common HLA-DRB1 (n = 20), -DQB1 (n = 11), -DPB1 (n = 8), -DQA1 (n = 5) and -DPA1 (n = 1) in our study cohort as explanatory variables. As explained above, HLA class II alleles with discordant typing results by HLA*LA and HLA-HD were excluded. We also excluded alleles with a MAF >0.2 to ensure homoscedasticity and HLA-DRA alleles due to the absence of polymorphisms of this gene in sequences encoding the peptide-binding groove (1), leaving a total of 45 alleles (**Table 3**) for inclusion in our association studies. The adjusted species-specific scores (a measure of antibody repertoire breadth) were used as response variables, while and age, sex and the first four genetic principal components were used as covariates. To further mitigate the potential effects of typing errors (e.g., at the sample level), we tested for associations with these 45 alleles as assigned by each HLA typing method independently and then prioritized associations identified regardless of the typing method used. As described above, stringent criteria were applied to test for strong (|β| ≥0.68) and significant associations, and we controlled for multiple testing using the Holm method (adjusted *P*-value ≤0.005). In total, we identified 30 associations (positive and negative) with the antibody responses to eight microbial species that were significant regardless of the HLA typing method used (**Figure 2**, round symbols) and 16 additional associations that reached significance with only one of the two HLA typing methods used (**Figure 2**, square symbols). We also defined a new feature for each tested HLA class II allele, namely the antimicrobial RR, which was calculated by dividing the number of significant associations of the antibody repertoire breadth to multiple microbial species by the total number of microbial species for which we had identified at least one association (see the Materials and Methods). The most robust positive associations (RR >0.3) were between HLA class II alleles DRB1*03:02, DRB1*07:01, DRB1*04:03, DQA1*04:01 and DQB1*03:02, and the breadth of the antibody repertoires against multiple microbial species, such as *S. pneumoniae*, *S. aureus*, *M. pneumoniae* and human metapneumovirus (HMPV), respectively. Negative associations where either restricted to only a single microbial species, or found only based on the results from one of the two HLA typing methods used here, and were therefore less robust (**Figure 2**).

**Table 3:** HLA class II alleles in the Qatar Biobank cohort (n = 800) considered for association studies.

			HLA*LA				HLA-HD		
Molecule	Gene	Allele	MAF	Hom. (n)	Het. (n)		MAF	Hom. (n)	Het. (n)
DP	DPA1	DPA1*02:02	0.063	4	93		0.070	4	104
DP	DPB1	DPB1*01:01	0.037	2	55		0.037	2	55
DP	DPB1	DPB1*02:01	0.181	30	230		0.181	30	230
DP	DPB1	DPB1*04:02	0.048	3	71		0.034	3	49
DP	DPB1	DPB1*09:01	0.018	1	27		0.018	1	27
DP	DPB1	DPB1*10:01	0.023	5	27		0.023	5	27
DP	DPB1	DPB1*13:01	0.054	9	68		0.052	9	65
DP	DPB1	DPB1*14:01	0.076	4	114		0.073	4	109
DP	DPB1	DPB1*17:01	0.026	0	42		0.024	0	39
DQ	DQA1	DQA1*01:01	0.073	8	100		0.032	2	47
DQ	DQA1	DQA1*01:03	0.056	3	83		0.056	3	83
DQ	DQA1	DQA1*02:01	0.176	34	214		0.176	34	214
DQ	DQA1	DQA1*03:01	0.148	29	179		0.116	20	145
DQ	DQA1	DQA1*04:01	0.017	0	27		0.016	0	25
DQ	DQB1	DQB1*03:01	0.116	10	166		0.103	8	149
DQ	DQB1	DQB1*03:02	0.123	22	153		0.123	22	153
DQ	DQB1	DQB1*03:03	0.011	0	18		0.011	0	18
DQ	DQB1	DQB1*04:02	0.025	0	40		0.025	0	40
DQ	DQB1	DQB1*05:01	0.063	6	88		0.063	6	89
DQ	DQB1	DQB1*05:02	0.154	37	172		0.154	37	172
DQ	DQB1	DQB1*05:03	0.014	0	22		0.014	0	22
DQ	DQB1	DQB1*06:01	0.024	0	39		0.024	0	39
DQ	DQB1	DQB1*06:02	0.049	4	70		0.049	4	70
DQ	DQB1	DQB1*06:03	0.040	3	58		0.040	3	58
DQ	DQB1	DQB1*06:04	0.029	3	40		0.029	3	40
DR	DRB1	DRB1*01:01	0.021	2	30		0.021	2	30
DR	DRB1	DRB1*01:02	0.014	0	23		0.014	0	23
DR	DRB1	DRB1*03:01	0.159	18	219		0.159	18	219
DR	DRB1	DRB1*03:02	0.016	1	23		0.016	1	23
DR	DRB1	DRB1*04:02	0.042	0	67		0.046	5	63
DR	DRB1	DRB1*04:03	0.054	0	86		0.058	6	81
DR	DRB1	DRB1*04:05	0.017	0	27		0.019	3	24
DR	DRB1	DRB1*07:01	0.151	0	241		0.181	35	219
DR	DRB1	DRB1*08:04	0.012	0	19		0.012	0	19
DR	DRB1	DRB1*10:01	0.026	0	41		0.026	0	41
DR	DRB1	DRB1*11:01	0.038	1	59		0.039	1	60
DR	DRB1	DRB1*11:04	0.029	1	44		0.029	1	44
DR	DRB1	DRB1*13:01	0.035	2	52		0.035	2	52
DR	DRB1	DRB1*13:02	0.040	4	56		0.040	4	56
DR	DRB1	DRB1*13:03	0.013	1	18		0.013	1	18
DR	DRB1	DRB1*15:01	0.056	4	82		0.056	4	82
DR	DRB1	DRB1*15:02	0.018	0	29		0.018	0	28
DR	DRB1	DRB1*15:03	0.012	0	19		0.012	0	19
DR	DRB1	DRB1*16:01	0.027	0	43		0.027	1	41
DR	DRB1	DRB1*16:02	0.105	12	144		0.114	24	135

MAF, minor allele frequency; hom., homozygous; het., heterozygous.

We also performed a regression analysis of the adjusted species-specific scores using the HLA-DRB1~DQA1~DQB1 haplotypes with a frequency ≥1% as explanatory variables (**Supplementary Table 4**). In this analysis, we identified 24 significant associations between nine haplotypes and the antibody repertoire breadth against eight microbial species, which were reproducible with either HLA typing method. In several instances, the strength of associations between species-specific antibody responses and haplotypes was higher than that when considering the respective alleles independently of a given haplotype, thus this analysis indicated the additive effects of multiple HLA class II alleles. Most notably, whereas the DRB1*07:01~DQA1*03:01~DQB1*03:02 haplotype was positively associated with antibody responses to HHV-1, HHV-4 and *S. pneumoniae*, the DRB1*01:01~DQA1*01:01~DQB1*05:01 haplotype was negatively associated with antibody responses to HHV-4, *S. pneumoniae*, *S. aureus*, and *H. pylori* (**Supplementary Table 7**).

### Associations between HLA-DRB1 genotypes and the antibody repertoire breadth against common microbial infections

Humans are diploid organisms and ultimately, it is likely that the combined effects of multiple HLA class II alleles encoded on both parental chromosomes defines the antibody binding repertoire of a given individual with a specific HLA class II genotype and diplotype. However, assessment of the role of all HLA-DQA1~DQB1~DRB1 diplotypes remains a challenge, primarily due to the extremely polymorphic nature of the classical HLA genes. Thus, studies of very large cohorts are required to achieve sufficiently sized groups with identical diplotypes for statistical comparison; this was not feasible in our cohort of 800 individuals. To overcome this issue, we tested for associations between specific HLA-DRB1 genotype groups and the breadth of the antibody repertoires against each of the common microbial species listed in **Table 2** and took advantage of the strong LD of the HLA class II loci (**Supplementary Figure 4**). Among the samples with concordant HLA typing results for *HLA-DRB1* by HLA*LA and HLA-HD (n = 697), we found 15 genotypes with sufficient sample sizes (n ≥10) for subsequent association studies (comprising 274 individuals, representing approximately 34% of our cohort). These genotypes included two groups of HLA-DRB1 homozygotes, namely HLA-DRB1*16:02 homozygotes and HLA-DRB1*03:01 homozygotes (**Table 3**). The remaining groups comprised HLA heterozygotes. Of note, homozygotes for the common HLA-DRB1*07:01 allele are found among the Qatari population, such as among transplant recipients and donors (Medhat Askar and Samira Abdulla Saleh, personal communication), but these were excluded from our analysis due to discordant typing results by HLA*LA and HLA-HD (**Supplementary Table 1**).

**Figure 3:**
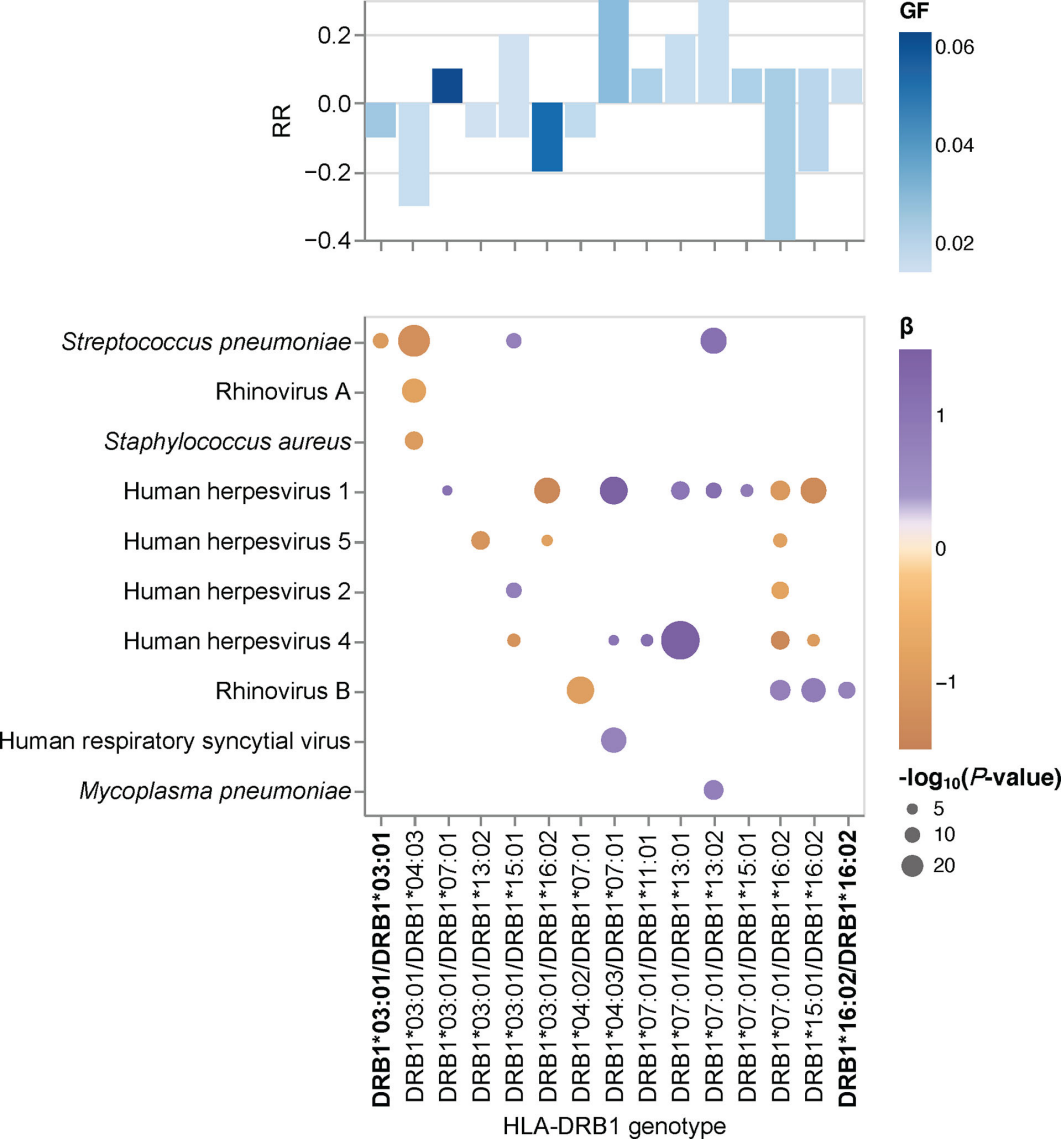
HLA-DRB1 genotype-specific effects on the antibody repertoire breadth against common microbial infections. Heatmap depicting significant associations (adjusted *P*-value <0.005) between specific HLA-DRB1 genotype groups and the breadth of the antibody repertoire against common microbial infections. The coefficient (β) and direction of association is indicated by a color gradient for each circle. The symbol size depicts the -log_10_(adjusted *P*-value) of the association. Bar plot depicting the anti-microbial response ratio (RR) for each HLA-DRB1 group. The genotype frequency (GF) is indicated by a color gradient for each bar. Groups with HLA-DRB1 homozygotes are labeled in bold.

To test for associations between the antibody repertoire breadth against common microbial species and specific *HLA-DRB1* genotypes, we performed a linear regression analysis of the adjusted species scores, this time using the HLA-DRB1 genotype group assignment described above as explanatory variables. Of the 15 HLA-DRB1 genotypes evaluated, we identified 31 significant associations with the antibody repertoire breadth against at least one of 10 microbial species (**Figure 3**). Highly robust positive associations (RR >0.3) were found for individuals carrying the DRB1*07:01 allele in combination with either DRB1*13:02 or DRB1*04:03. Notably, only one significant association was found for each of the HLA-DRB1*03:01 homozygote and HLA-DRB1*16:02 homozygote groups. In accordance with the results of our association studies at the allele and haplotype levels, associations between HLA-DRB1 genotypes and the antibody repertoire breadth were observed mainly for a limited number of microbial species, including human herpesviruses (HHV-1, HHV-2, HHV-4, HHV-5), ‘common cold’ RNA viruses (HRV-A, HRV-B, HRSV) and bacterial species, such as *S. pneumoniae*, *S. aureus* and *M. pneumoniae* (**Figure 3**).

### Associations of HLA-DRB1 genotypes with specific antigens

**Figure 4:**
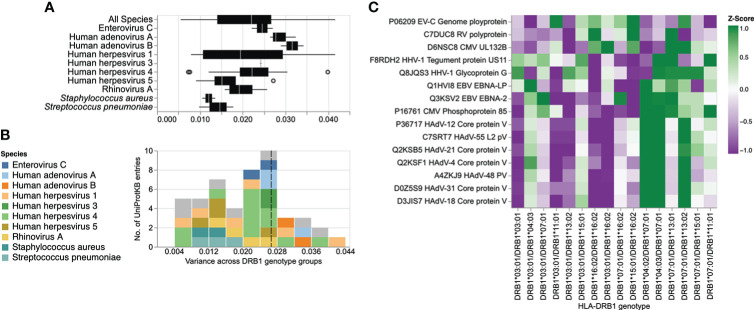
Differential enrichment analysis of antigenic peptide-antibody interactions among different HLA-DRB1 groups. **(A)** Box plot illustrating the distribution of variance in the mean peptide enrichment frequency per UniProtKB entry of all, or selected, microbial species, and across different HLA-DRB1 genotype groups. **(B)** Histogram of variance in the mean peptide enrichment frequency per UniProtKB entry, as shown in **(A)**. High variance captures protein antigens of microbial species that exhibit a comparatively different peptide enrichment profile across HLA-DRB1 genotype groups. The dashed line in **(B)** indicates the boundary of the upper 25^th^ percentile (variance ≥ 0.026). UniProtKB entries for which the antibody-antigen interactions showed the highest variance across different DRB1 groups are color-coded by species. **(C)** Heatmap showing the antibody binding profile of selected microbial antigens across different HLA-DRB1 groups, with hierarchical clustering. Each row is a protein (UniProtKB entry) with a variance ≥ 75% quantile in the mean peptide enrichment frequency as shown in **(B)**; each column represents a HLA-DRB1 genotype group. The color gradient represents the mean enrichment score (Z-score) of antigenic peptides per protein antigen and HLA-DRB1 genotype group.

Finally, we sought to assess the effect of specific HLA-DRB1 alleles and genotypes at the antigen level. The gene products of advantageous HLA alleles and genotypes may not only be capable of presenting a broader array of pathogen-derived peptides than risk alleles and genotypes, but may also enhance the peptide-binding specificity and presentation of selected antigenic regions (i.e., epitopes) (42). To explore the effect of HLA-DRB1 genotypes (n = 16) on antibody binding specificities to common microbial antigens, we first filtered for peptide antigens that were significantly enriched in at least two samples in our cohort and were also differentially enriched across the different DRB1 groups described above, by using Fisher’s exact test [-log_10_(*P*-value) ≥2.3] and an OR of ≥2 or ≤-2 as the cut-off. Interestingly, most of the variance among the retained DEP antigens was due to antibodies targeting proteins of a relatively few microbial species, most notably HHV-1, HHV-3, HHV-4, HHV-5, HAdVs A and B, HRV-A, EV-C, *S. pneumoniae*, and *S. aureus* (**Figures 4A, B**). We then filtered for protein antigens for which the DEPs showed high variance (above the 75^th^ quartile) across the different DRB1 groups (for details see the Materials and Methods section). Following the application of these stringent filter criteria, 15 protein antigens were retained, representing 10 microbial species, most notably HHVs and HAdVs (**Figure 4C**). Among these microbial antigens, we found considerable variance in the antibody specificities targeting a variety of HHV proteins, including the glycoprotein G and tegument protein US11 of herpes simplex virus 1 (HSV-1, species HHV-1), the Epstein–Barr virus nuclear antigens (EBNA)-2 and 5 (EBV, species HHV-4), as well as phosphoprotein 85 of human cytomegalovirus (CMV, species HHV-5) (**Figures 4B, C** and **Supplementary Figures 5A–C**), which was consistent with our findings at the species level. EBNA-2 for example, was frequently targeted by individuals who were heterozygous for HLA-DRB1*07:01 and HLA-DRB1*13:01 (**Figures 4C**, **Supplementary Figure 5B**), a genotype that was also strongly and positively associated with the breadth of the antibody repertoire against EBV (HHV-4) (**Figure 3**). In contrast, individuals heterozygous for HLA-DRB1*03:01 and HLA-DRB1*13:02 largely lacked an antibody response to phosphoprotein 85 of CMV (**Figures 4C**, **Supplementary Figure 5C**) and exhibited a negative association with the breadth of the antibody repertoire against CMV (HHV-5) (**Figure 3**). A multiple sequence alignment of these HHV antigens by Clustal Omega did not reveal linear amino acid sequence similarities (not shown), indicating that these antigens are targeted by antibodies with distinct specificities owing to multiple HLA class II alleles. We also found that individuals with certain HLA-DRB1 genotypes (e.g., HLA-DRB1*04:01/HLA-DRB1*07:01, HLA-DRB1*04:03/HLA-DRB1*07:01 or HLA-DRB1*13:01/HLA-DRB1*07:01) had antibodies that frequently targeted antigenic peptides of different HAdV species (**Figure 4C** and **Supplementary Figures 5D–F**). All these peptides showed a high degree of amino acid similarity and resembled a region of an orthologous core protein expressed by the different species (**Supplementary Figure 6**), suggesting the existence of similar antibody specificities that may also cross-react with antigens of the other HAdV species.

## Discussion

In this study, we employed a systematic and unbiased approach to explore the relative contribution of germline genetic variation in classical HLA class II genes among the general adult population to human antibody responses, including antibody specificities to 48 common human-tropic pathogenic microbial species. By applying a high-throughput method for large-scale antibody profiling to a well-defined cohort of mostly Qatari nationals sharing genetic material due to the high frequency of consanguineous unions, we dissected the overall effect of zygosity for classical HLA class II genes, as well as the effects associated with specific HLA class II alleles, haplotypes and genotypes, on the antimicrobial antibody repertoire breadth and antibody specificity with unprecedented resolution.

Our results provide indirect evidence that heterozygosity in classical HLA class II genes confers a selective advantage in humans. Heterozygote advantage has been proposed as one of the main mechanisms that has driven HLA allelic diversity and resistance to infection during human evolution. However, empirical evidence from human studies has been sparse (1, 5, 43, 44). We demonstrate that overall (i.e., irrespective of the allele), individuals heterozygous for *HLA-DRB1*, *-DQB1* and -*DQA1* have a broader antibody repertoire against a variety of viral and opportunistic bacterial pathogens, including HHV-6B, HHV-7, HHV-8, human parvovirus B19, HCoV-229E, HCoV-HKU1, IBV, ICV, human parainfluenza virus 1, rotavirus A, alphapapillomavirus 9, *H. pylori*, and *M. pneumoniae*, when compared to their homozygous peers. We also found heterozygosity in the less polymorphic *HLA-DPB1* gene to be negatively associated with the breadth of the antibody repertoire against HHV-4, HHV-8, HAdV-C, ICV and *S. pneumoniae*, as well as a negative association between antibody responses against EV-B and HHV-3, and heterozygosity in *HLA-DRB1*, *-DQA1* and *-DQB1*. It is unclear whether the latter observation reflects a disadvantage for heterozygotes, or rather, more frequent reactivations of this latent virus (i.e., shingles) among *HLA-DRB1*, *-DQA1* and *-DQB1* homozygotes. The negative association between heterozygosity in *HLA-DPB1* and antibody responses against several microbial species is surprising and more difficult to reconcile with a heterozygote advantage. One may speculate that heterozygosity in this gene could present a disadvantage in the context of some common viral and bacterial infections and at the same time represent an advantage in another context, such as autoimmunity or allergy (14). Alternatively, other selection mechanisms may have had a more prominent contribution to the genetic diversity of HLA-DPB1, including negative frequency-dependent selection (1, 5). In accordance with a heterozygote advantage of classical HLA class II loci, our study of associations between HLA-DRB1 genotypes and specific antigens revealed that the two groups comprising HLA-DRB1*16:02 homozygotes and HLA-DRB1*03:01 homozygotes exhibited poor antibody responses against antigens of a variety of HAdV and HHV species when compared to their heterozygous peers. Previous studies have demonstrated a heterozygote advantage for classical HLA class II genes in the context of human HBV infection (43), HBV-associated hepatocellular carcinoma (45), and in patients with ulcerative colitis (44). Moreover, a heterozygote advantage has also been reported by Hraber *et al.* (46) in the context of HCV infection but this could not be confirmed in an independent study by Shaheen *et al.* (47). A heterozygote advantage in classical HLA class I loci has been documented in the context of HIV infection, as this virus produces escape variants during chronic infection at a considerable frequency (1). Maximum HLA heterozygosity of the classical HLA class I genes *HLA-A*, *-B* and *-C* has been associated with delayed disease onset among HIV-1 infected patients, whereas individuals who were homozygous for one or more loci progressed rapidly to AIDS and death (42, 48). Other well-known examples of heterozygote advantage include the recessive disease-causing variants underlying sickle-cell anemia, with one copy of the HbS allele shown to protect heterozygotes from severe forms of malaria (49). Interestingly, an in silico analysis by Sellis et al. (50) suggested that a substantial proportion of host adaptive mutations occurring during human and vertebrate evolution confer a heterozygote advantage, as rapidly changing environments and genetic variation produce a diversity advantage in diploid organisms that allows them to remain better adapted compared with haploids, despite the fitness disadvantage associated with the occurrence of rare homozygotes (50).

Our findings also demonstrate that multiple alleles of the classical HLA class II genes (i.e., HLA-DRB1, -DQA1 and -DQB1) can have an additive effect on the antibody repertoire against microbial pathogens, although in some instances, alleles from the orthologous HLA class II genes may also have opposing effects (e.g., in individuals carrying the DRB1*16:02~DQA1*02:01~DQB1*05:02 haplotype). Our results therefore support the concept that viral infections, along with other infectious diseases, have helped to maintain strong immunity and resistance to common infections during human evolution by promoting diversity in HLA class II alleles and consequently, in B cell-mediated antibody responses (51). The reasons why HLA diversity remains relatively low at the individual (host) level have been debated since expression of more HLA molecules or molecular variants by a given individual, which may arise through gene duplication events that have occurred throughout vertebrate evolution, would theoretically allow the binding and presentation of an even a broader spectrum of antigens, thereby enhancing immunity to infections (it should be noted that this may be the case for some individuals with haplotypes that express additional functional DRB genes that were not present in our study cohort) (5). The associated trade-off effects appear to be the most plausible explanation. Indeed, certain HLA alleles have been shown to play a protective role in the context of certain infectious diseases, while at the same time being associated with an increased risk for autoimmune diseases (5, 13, 52). In this regard, it should be noted that the HLA-DRB1*03:01 allele, which was relatively common in our study cohort (MAF = 0.159), has been reported to be a risk allele for autoimmune hepatitis (AIH) (53). AIH may develop not only after hepatitis A, B or C infections, but also following infection with more common pathogens such as HSV-1, EBV, or measles virus. The prevalence of AIH in the general adult population in this study remains unknown.

Interestingly, using our unbiased, large-scale screen and in-depth analysis of antibody specificities against 48 microbial species, we predominantly and repeatedly identified positive associations with antibody responses against members of the *Herpesviridae* family [such as HSV-1 (HHV-1), VZV (HHV-3), EBV (HHV-4), CMV (HHV-5), and roseolavirus (HHV-6B)], *Picornaviridae* (including HRV-A and -B, EV-A, -B and -C), *Paramyxoviridae* (e.g., HRSV, and HMPV), and *Adenoviridae* (HAdV-C), as well as opportunistic bacterial pathogens that frequently colonize the upper airways of humans but are typically innocuous (e.g., *S. aureus*, *S. pneumoniae* and *M. pneumoniae*). This raises the question of whether these microbial species have also played a critical role during hominine evolution by driving genetic diversity in the classical HLA class II loci. Recent advances in microbial genetics techniques enabling molecular clock analyses suggest that, although phylogenetically diverse, many, if not all of these species have evolved in very close association with their human host, some of them (e.g., HSV-1) for millennia; similar findings were obtained for their counterparts infecting primates or other vertebrates (51). Indeed, although cross-species transmissions have occurred in the more recent past, it is becoming increasingly evident that most human pathogens originated long before the Neolithic era (54). A commonly stated hypothesis is that pandemic outbreaks of major human infectious diseases (e.g., influenza, hepatitis, tuberculosis, malaria, leishmaniasis, and schistosomiasis) that occurred in the more recent (i.e., the post-Neolithic) past and causing considerable morbidity and mortality, have been major driving forces of HLA genetic diversity. While this may be true based on the identification of several positive and negative HLA/MHC associations with these diseases (13), the role of other human infectious agents, particularly those that have co-evolved with their human host for much longer periods, should not be neglected simply on the basis that they cause no, or only mild, clinical disease in most cases of (modern) human infection. Even herpesviruses, such as HSV-1, EBV or CMV, which are most commonly acquired in early life or childhood, can cause fatal disease in rare cases, either following primary infection of genetically susceptible individuals (55), or reactivated infections in patients with cancer, autoimmune diseases or other comorbidities (56). Moreover, infections can have more subtle effects on human reproductive fitness. The effects of these ‘modern human pathogens’ on our hominine ancestors and phylogenetically closest relatives (i.e., archaic humans, such as Neanderthals and Denisovans) that are extinct today are also unknown.

It is also important to highlight the limitations of our study. First and foremost, HLA typing methods from 30× WGS data are error-prone, leading to discordant typing results depending on the gene, algorithm, and resolution (40, 41) (**Supplementary Figure 1**), which may give rise to spurious associations. We attempted to overcome this limitation by leveraging two of the most popular methods, namely HLA*LA and HLA-HD, along with an up-to-date allele dictionary from IPD-IMGT/HLA database. Although we observed relatively high concordance across different resolutions, we only considered alleles with an error rate <5% in calling and also excluded rare alleles (MAF <0.01) due to sample size limitations in our association studies. Moreover, with our large-scale antibody screening approach, we were primarily able to assess antibody specificities and repertoires to linear epitopes of protein antigens, predominantly of human-tropic viruses. Although there is evidence these include neutralizing and non-neutralizing antibodies (35), further investigations are required to elucidate the extent to which these genetic and associated immune phenotypic differences affect clinical outcomes of infection, either by long-term longitudinal studies of even larger human cohorts, or a case-control study of selected diseases. It is highly plausible that positive associations between specific HLA types and broad antibody responses to common pathogens represent a selective advantage (assuming that individuals with stronger antibody responses are likely able to better recognize and clear infections) and independent studies have demonstrated that variants in the HLA class II region (as well as age) are strong predictors of antibody responses to natural infection and vaccination (18). However, broad antibody repertoires may also be a consequence of chronic or a more recent/frequent acute infection in some individuals. Notwithstanding these limitations, our study provides strong evidence in support of heterozygote advantage and the additive effect of multiple alleles from orthologous HLA class II genes among humans, most notably by demonstrating that (i) zygosity of the classical HLA class II genes is a strong predictor of antibody responses to common human pathogens; (ii) several haplotypes are stronger predictors of antibody responses than their respective alleles alone; and (iii) DRB1 heterozygotes with alleles also observed in DRB1 homozygotes exhibit stronger antibody responses to specific microbial antigens than their homozygous counterparts. The extent to which these findings affect clinical outcomes of natural infection and perhaps also routine vaccination [which is thought to confer protection mainly through the induction of antibodies; reviewed in (57)], remains to be investigated.

## Data availability statement

All processed data are available in the manuscript or the supplementary materials. Raw reads from PhIP-Seq are available in the NCBI Sequence Read Archive (Accession: PRJNA685111 and PRJNA688708). Python in-house scripts used in this study are available upon request. The pipeline for processing the PhIP-Seq data has been published previously (19). Raw WGS data of the study participants are accessible through the Qatar Genome Programme (https://qatargenome.org.qa; e-mail: genome@qf.org.qa). Processed genetic data (i.e., output data from the HLA type inference using HLA*LA and HLA-HD), relevant covariates used for our association models and the adjusted species score values that we used as a quantitative measure of the antibody repertoire breadth can be found in Supplementary Tables 1, 2 and 5, respectively. For additional individual phenotypic data, requests should be made directly to QBB (https://www.qatarbiobank.org.qa). The data management infrastructure of QBB has been described previously (58).

## Ethics statement

The studies involving human participants were reviewed and approved by the Institutional Review Board of Sidra Medicine and the Institutional Review Board of Qatar Biobank. The patients/participants provided their written informed consent to participate in this study.

## Author contributions

NM conceived the study and supervised the project. MR and FA designed and performed experiments. TK developed the data analysis tools for the association studies and differential enrichment analysis. TK, NM, and IA analyzed and interpreted the data. PJ co-supervised the HLA variant analysis. NM and TK wrote the paper. All authors have seen and approved the manuscript, which has not been accepted or published elsewhere.

## Funding

This work was supported in part by a grant from the Qatar National Research Fund (PPM1-1220-150017) and funds from Sidra Medicine (SDR400127).

## Acknowledgments

We thank the QBB study participants who provided samples and data for this study. We also thank Qatar Genome and the QBB management and staff, in particular Nahla Afifi, Said I. Ismail and Elizabeth Jose, for allowing us to access and analyze QBB/QGP samples and data; the Integrated Genomics Services team of Sidra Genomics for generating and processing WGS data of study participants; Stephen Elledge (Brigham and Women’s Hospital, Harvard University Medical School) for kindly providing the VirScan phage library used in this study and for his early discussions related to this work; Tomasz Kula (Brigham and Women’s Hospital, Harvard University Medical School) and Benjamin Larman (Johns Hopkins School of Medicine) for their advice on technical aspects related to the PhIP-Seq experiments; and Jessica Tamanini (Insight Editing London) for proofreading and editing the manuscript.

## Conflict of interest

The authors declare that the research was conducted in the absence of any commercial or financial relationships that could be construed as a potential conflict of interest.

## Publisher’s note

All claims expressed in this article are solely those of the authors and do not necessarily represent those of their affiliated organizations, or those of the publisher, the editors and the reviewers. Any product that may be evaluated in this article, or claim that may be made by its manufacturer, is not guaranteed or endorsed by the publisher.

## Supplementary material

Supplementary Table 1 HLA genotypes for each study subject inferred by using HLA*LA and HLA-HD.

Supplementary Table 2 Covariates of association models.

Supplementary Table 3 List of HLA class II alleles, including allele frequencies, number of homozygous individuals and number of heterozygous individuals in our cohort of 800 individuals, as determined by HLA*LA and HLA-HD.

Supplementary Table 4 HLA-DRB1~DQA1~DQB1 haplotypes in the study cohort (n = 800) with an estimated haplotype frequency ≥1%.

Supplementary Table 5 Adjusted species score values.

Supplementary Table 6 Results of a multivariate association analysis of age and sex with the antibody repertoire breadth against selected species (n = 48).

Supplementary Table 7 Combined effects of HLA-DRB1, -DQA1 and -DQB1 alleles as haplotypes.

Supplementary Figure 1 Concordance between HLA typing methods and allele selection for association studies. **(A, B)** Dot plots depict the allele frequencies (AF) of classical HLA class II genes, as determined by HLA*LA and HLA-HD from our cohort of 800 individuals **(A)**, including selected alleles considered for association studies **(B)**. Gray symbols depict alleles with an AF of <0.01 or >0.2 and alleles with discordant typing results (|AF_HLA-HD_ - AF_HLA-LA_| < 0.05), which were excluded from the association studies. Colored symbols depict alleles considered for association studies. The shape of the symbol indicates the encoded molecule. **(C)** Percentage of concordance between the two HLA typing methods for each locus at different resolution (allele group, 1-field; protein sequence, 2-field; maximum resolution, G group). **(D)** Overlay bar chart depicting the number of alleles for each of the classical HLA class II genes investigated, as determined by HLA*LA and HLA-HD from our cohort of 800 individuals (grey bars), including selected alleles considered for association studies (blue bars).

Supplementary Figure 2 Genetic structure of the study cohort and relationship of genetic principal components with heterozygosity rates, participant nationality and classical HLA class II alleles. **(A)** Comparison of individual heterozygosity rates among the study cohort (QBB, n = 800) and reference data of the 1000 Genomes Project (1KGP, n = 3,881). **(B)** Genetic principal component analysis of the study cohort depicting the distribution of individuals according to their heterozygosity rates. **(C)** Genetic principal component analysis of the study cohort (shown as diamonds) depicting the distribution of individuals according to nationality. Reference data of the 1000 Genomes Project are superimposed (shown as circles and color-coded based on ethnicity). AFR, African; EUR, European, SAS, South Asian, AMR, American, EAS, East Asian.

Supplementary Figure 3 Multiple sequence alignment and phylogenetic tree of *HLA-DRB1* gene products. **(A)** Alignment of all HLA-DRB1 alleles analyzed in this study with the reference sequence (NP_002115.2) using MUSCLE aligner in JalView (v 2.11.1.4). The color in **(A)** highlights percentage identity. **(B)** Cladogram of the sequences shown in **(A)**, with the real branch length indicated by the number shown for each branch. Sequences were retrieved from the IPD-IMGT/HLA database (https://www.ebi.ac.uk/ipd/imgt/hla/allele.html).

Supplementary Figure 4 Entropy-based linkage disequilibrium (eLD) index of classical HLA class I and II genes. Pairwise LD index of *ϵ* among the classical HLA alleles in our study cohort (n = 800) were evaluated using eLD.

Supplementary Figure 5
**(A-F)** Comparative antigenicity profiles of selected microbial antigens across DRB1 genotype groups. Only representative DRB1 genotype groups with high variance in the Z-score values are shown. In the heatmap plots, each row is a peptide tiling across the indicated protein; each column represents a random sample of the selected DRB1 genotype groups (at least 10 samples per group are shown). Individuals of the same group are indicated with a color bar (bottom). The color intensity of each cell corresponds to the -log_10_(*P*-value), which was used as a measure of enrichment for a peptide in a sample. Greater values indicate stronger antibody responses; a -log_10_(*P*-value) ≥2.3 was considered to indicate statistical significance.

Supplementary Figure 6 Multiple sequence alignment (Clustal Omega) of the L2 gene products (Core protein V) of different HAdV species shown in . Red arrows and the box indicate the UniProtKB entries and antigenic region corresponding to the peptide tiles with strong antibody responses shown in **Supplementary Figures 5D, F**.
