# Supplementary material for: Human leukocyte antigen class II gene diversity tunes antibody repertoires to common pathogens
Source: Front Immunol. 2022 Aug 8;13:856497. doi: 10.3389/fimmu.2022.856497 (PMC9393332; doi:10.3389/fimmu.2022.856497)
Supplement: Supplementary Table 1 — HLA genotypes for each study subject inferred by using HLA*LA and HLA-HD. [file DataSheet_1.zip › Supplementary Material/Supplementary Table 4.PDF]

HLA-DRB1~DQA1~DQB1 haplotype	HLA*LA (Sample_Count)	HLA*LA (HF)	HLA-HD (Sample_Count)	HLA-HD (HF)
DRB1*01:01~DQA1*01:01~DQB1*05:01	21	0.013125	21	0.013125
DRB1*03:01~DQA1*01:02~DQB1*02:01	71	0.044375	71	0.044375
DRB1*03:01~DQA1*02:01~DQB1*02:01	44	0.0275	43	0.026875
DRB1*03:01~DQA1*03:01~DQB1*02:01	35	0.021875	23	0.014375
DRB1*03:01~DQA1*05:01~DQB1*02:01	70	0.04375	70	0.04375
DRB1*04:02~DQA1*03:01~DQB1*03:02	15	0.009375	20	0.0125
DRB1*04:03~DQA1*03:01~DQB1*03:02	14	0.00875	19	0.011875
DRB1*07:01~DQA1*01:02~DQB1*02:01	45	0.028125	ND	ND
DRB1*07:01~DQA1*02:01~DQB1*02:01	69	0.043125	8	0.005
DRB1*07:01~DQA1*03:01~DQB1*03:02	36	0.0225	40	0.025
DRB1*07:01~DQA1*05:01~DQB1*02:01	40	0.025	ND	ND
DRB1*15:01~DQA1*01:02~DQB1*05:02	18	0.01125	18	0.01125
DRB1*16:02~DQA1*01:02~DQB1*05:02	55	0.034375	64	0.04
DRB1*16:02~DQA1*02:01~DQB1*05:02	17	0.010625	19	0.011875
DRB1*16:02~DQA1*05:01~DQB1*05:02	51	0.031875	39	0.024375
DRB1*07:01~DQA1*01:02~DQB1*02:02	ND	ND	46	0.02875
DRB1*07:01~DQA1*02:01~DQB1*02:02	ND	ND	93	0.058125
DRB1*07:01~DQA1*05:01~DQB1*02:02	ND	ND	41	0.025625

HF, Haplotype Frequency; ND, not detected
