# Supplementary material for: Human leukocyte antigen class II gene diversity tunes antibody repertoires to common pathogens
Source: Front Immunol. 2022 Aug 8;13:856497. doi: 10.3389/fimmu.2022.856497 (PMC9393332; doi:10.3389/fimmu.2022.856497)
Supplement: Supplementary Table 1 — HLA genotypes for each study subject inferred by using HLA*LA and HLA-HD. [file DataSheet_1.zip › Supplementary Material/Supplementary Table 6.PDF]

| Feature[A] | Species[B] | Coefficient of Association [95% Confidence Interval] |
|---|---|---|
| Age | Human herpesvirus 5 | 1.41 [0.93,1.89] |
| | Human herpesvirus 4 | 1.32 [0.65,2.0] |
| | Human herpesvirus 1 | 1.2 [0.68,1.72] |
| | Human herpesvirus 2 | 1.1 [0.54,1.66] |
| | Enterovirus A | -0.75 [-0.98,-0.52] |
| | Human herpesvirus 6B | -0.76 [-1.03,-0.49] |
| | Rhinovirus B | -0.91 [-1.27,-0.55] |
| | Human adenovirus C | -0.95 [-1.22,-0.68] |
| | Rhinovirus A | -1.1 [-1.39,-0.81] |
| | *Streptococcus pneumoniae* | -1.36 [-1.77,-0.95] |
| Female | *Streptococcus pneumoniae** | 3.33 [3.1,3.55] |
| | *Staphylococcus aureus* | 2.68 [2.42,2.94] |
| | Human herpesvirus 4* | 2.46 [2.1,2.83] |
| | Rhinovirus B | 2.28 [2.09,2.47] |
| | Human herpesvirus 5 | 1.83 [1.57,2.09] |
| | Rhinovirus A | 1.81 [1.66,1.97] |
| | Human adenovirus C | 1.62 [1.47,1.77] |
| | Human herpesvirus 6B | 1.39 [1.25,1.54] |
| | Human respiratory syncytial virus | 1.24 [1.09,1.39] |
| | Human herpesvirus 1 | 1.18 [0.9,1.46] |
| | *Mycoplasma pneumoniae* | 1.09 [0.91,1.28] |
| | Enterovirus B | 1.08 [0.98,1.18] |
| | Human herpesvirus 7 | 1.05 [0.85,1.25] |
| | Enterovirus A | 0.98 [0.86,1.1] |
| | Enterovirus C | 0.93 [0.8,1.05] |
| | Human herpesvirus 3 | 0.8 [0.68,0.93] |
| | Human parainfluenza virus 3 | 0.74 [0.58,0.91] |
| | Influenza A virus | 0.74 [0.62,0.86] |
| | *Helicobacter pylori** | 0.68 [0.48,0.89] |
| Male | *Streptococcus pneumoniae* | 3.13 [2.89,3.37] |
| | *Staphylococcus aureus* | 2.61 [2.34,2.89] |
| | Rhinovirus B | 2.47 [2.27,2.68] |
| | Human herpesvirus 4 | 2.19 [1.8,2.57] |
| | Rhinovirus A* | 2.03 [1.87,2.2] |
| | Human herpesvirus 5 | 1.66 [1.38,1.93] |
| | Human adenovirus C | 1.49 [1.34,1.65] |
| | Human herpesvirus 1 | 1.31 [1.01,1.61] |
| | Human respiratory syncytial virus | 1.23 [1.07,1.39] |
| | Human herpesvirus 6B | 1.21 [1.06,1.37] |
| | *Mycoplasma pneumoniae* | 1.15 [0.96,1.35] |
| | Enterovirus B | 1.13 [1.02,1.23] |
| | Human herpesvirus 7 | 1.07 [0.86,1.29] |
| | Enterovirus A | 1.05 [0.92,1.18] |
| | Enterovirus C | 1.03 [0.9,1.17] |
| | Human herpesvirus 3 | 0.83 [0.7,0.96] |
| | Influenza A virus | 0.81 [0.68,0.93] |
| | Human parainfluenza virus 3 | 0.72 [0.55,0.89] |
| | Influenza B virus* | 0.68 [0.57,0.8] |

[A] Normalized age, male and female sex were used as independent explanatory variables. Only results with a significant association (P-value <= 0.0001) are shown; [B] Adjusted species score values (response variables) were used as a measure of antibody repertoire breadth against the listed species. The star symbol (*) indicates significant associations only found with one sex (independent variable) or stronger associations with one sex (i.e., if the difference in beta values for each sex and species was greater than the 75th percentile of the distribution of the differences in beta values for all species (i.e., $|beta_{Female}-beta_{Male}| > 0.197$).
