# Supplementary figures and images for: Human leukocyte antigen class II gene diversity tunes antibody repertoires to common pathogens
Source: Front Immunol. 2022 Aug 8;13:856497. doi: 10.3389/fimmu.2022.856497 (PMC9393332; doi:10.3389/fimmu.2022.856497)

# Supplementary Figure 1

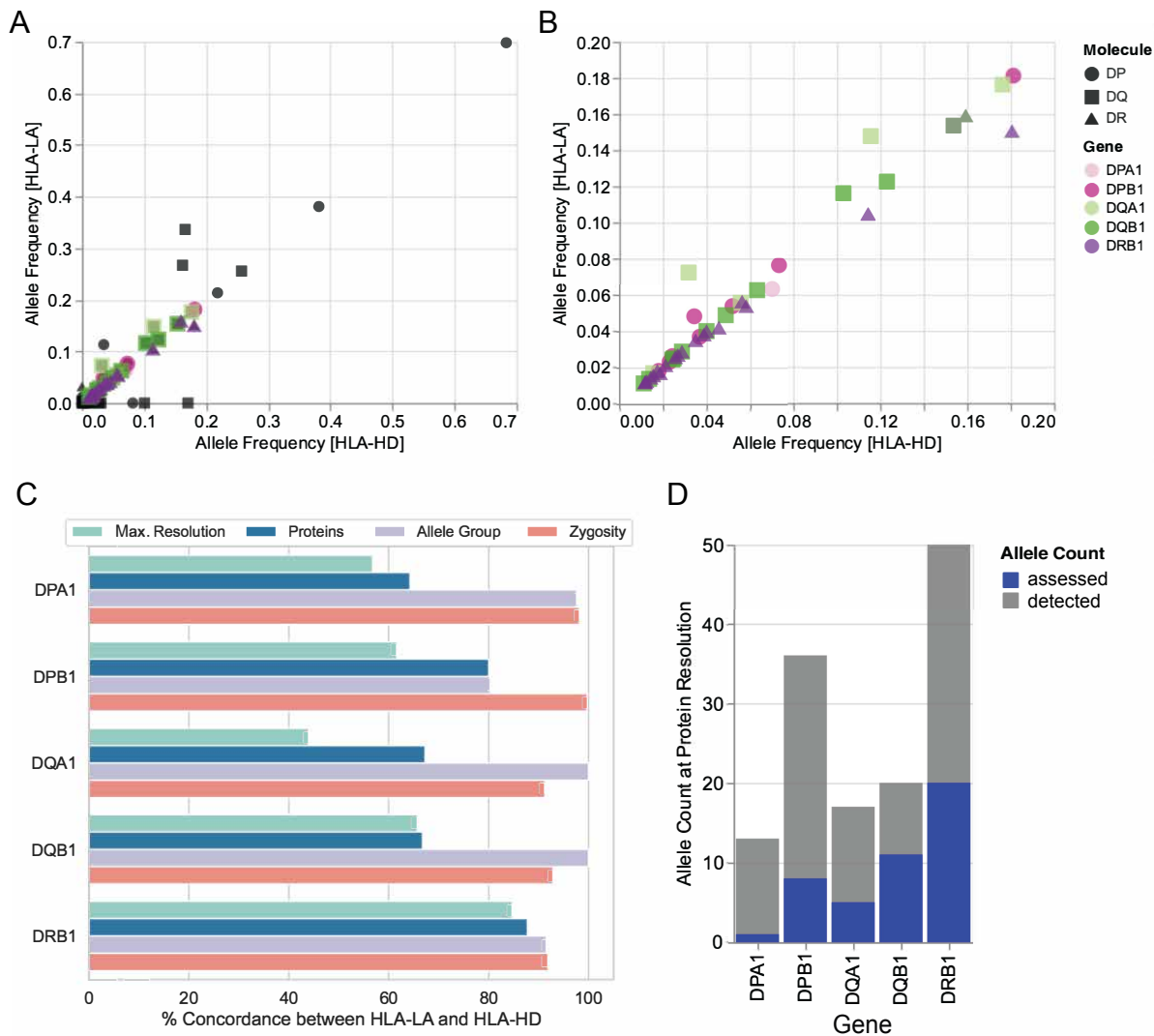

Supplement: Supplementary Table 1 — HLA genotypes for each study subject inferred by using HLA*LA and HLA-HD. [file DataSheet_1.zip › Supplementary Material/Supplementary Figure 1.PDF]

## Supplementary Figure 2

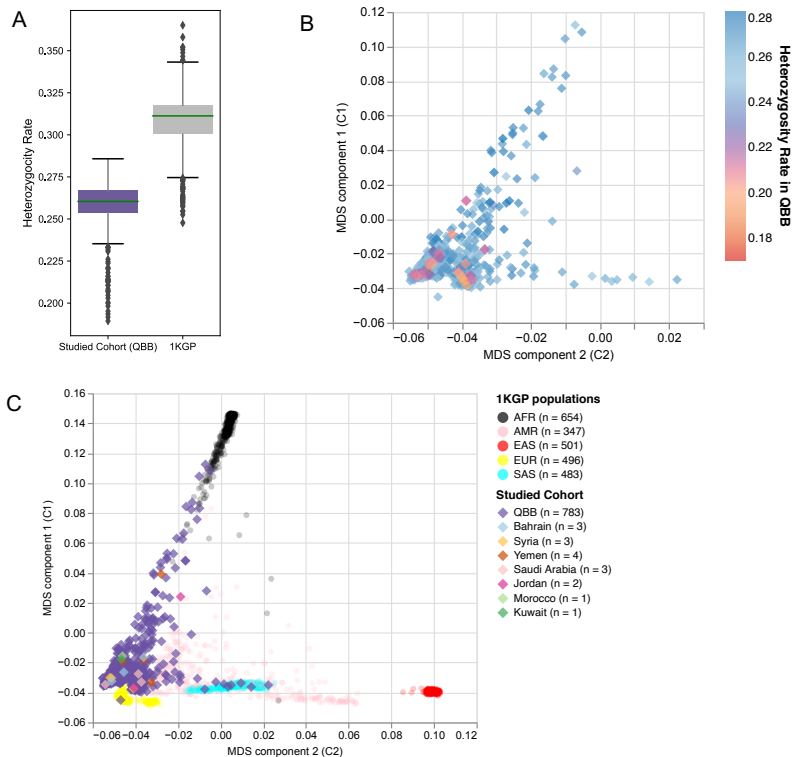

Supplement: Supplementary Table 1 — HLA genotypes for each study subject inferred by using HLA*LA and HLA-HD. [file DataSheet_1.zip › Supplementary Material/Supplementary Figure 2.PDF]

### Supplementary Figure 3

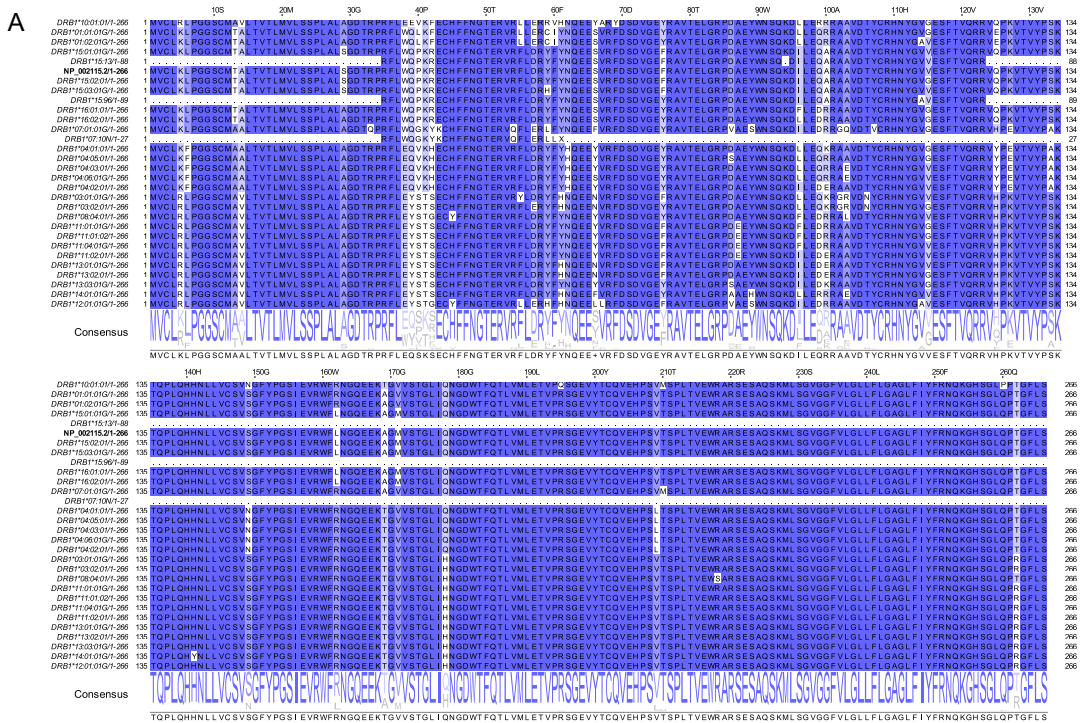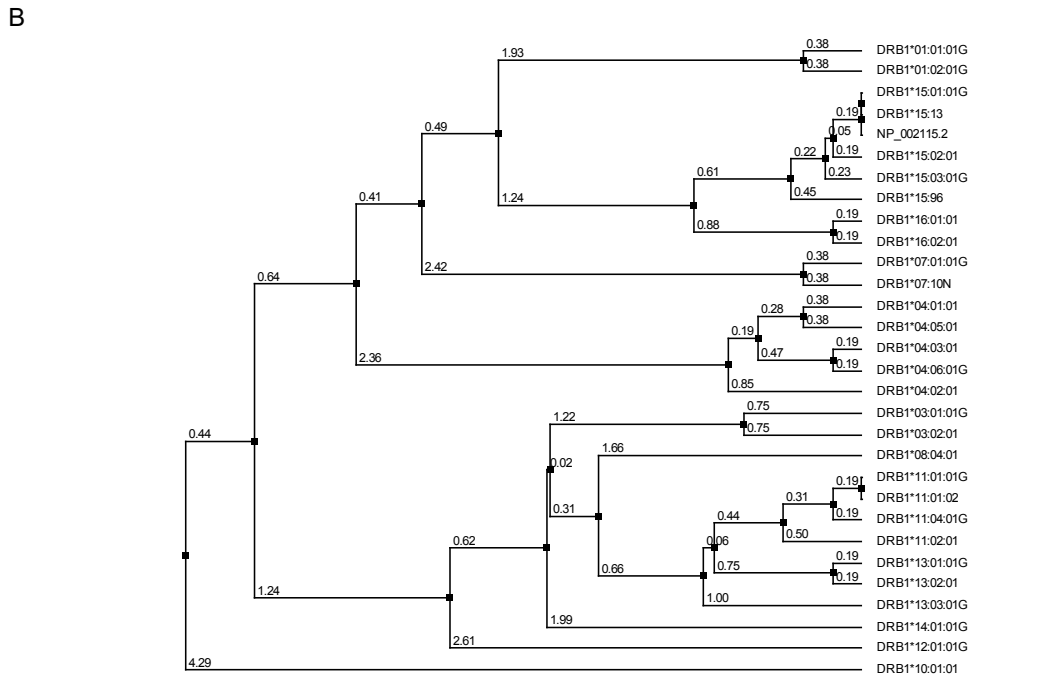

Supplement: Supplementary Table 1 — HLA genotypes for each study subject inferred by using HLA*LA and HLA-HD. [file DataSheet_1.zip › Supplementary Material/Supplementary Figure 3.PDF]

Supplementary Figure 4

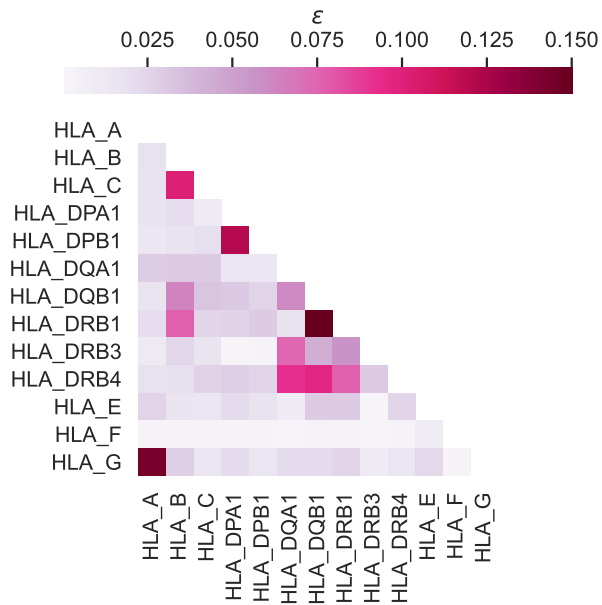

Supplement: Supplementary Table 1 — HLA genotypes for each study subject inferred by using HLA*LA and HLA-HD. [file DataSheet_1.zip › Supplementary Material/Supplementary Figure 4.PDF]

Supplementary Figure 5

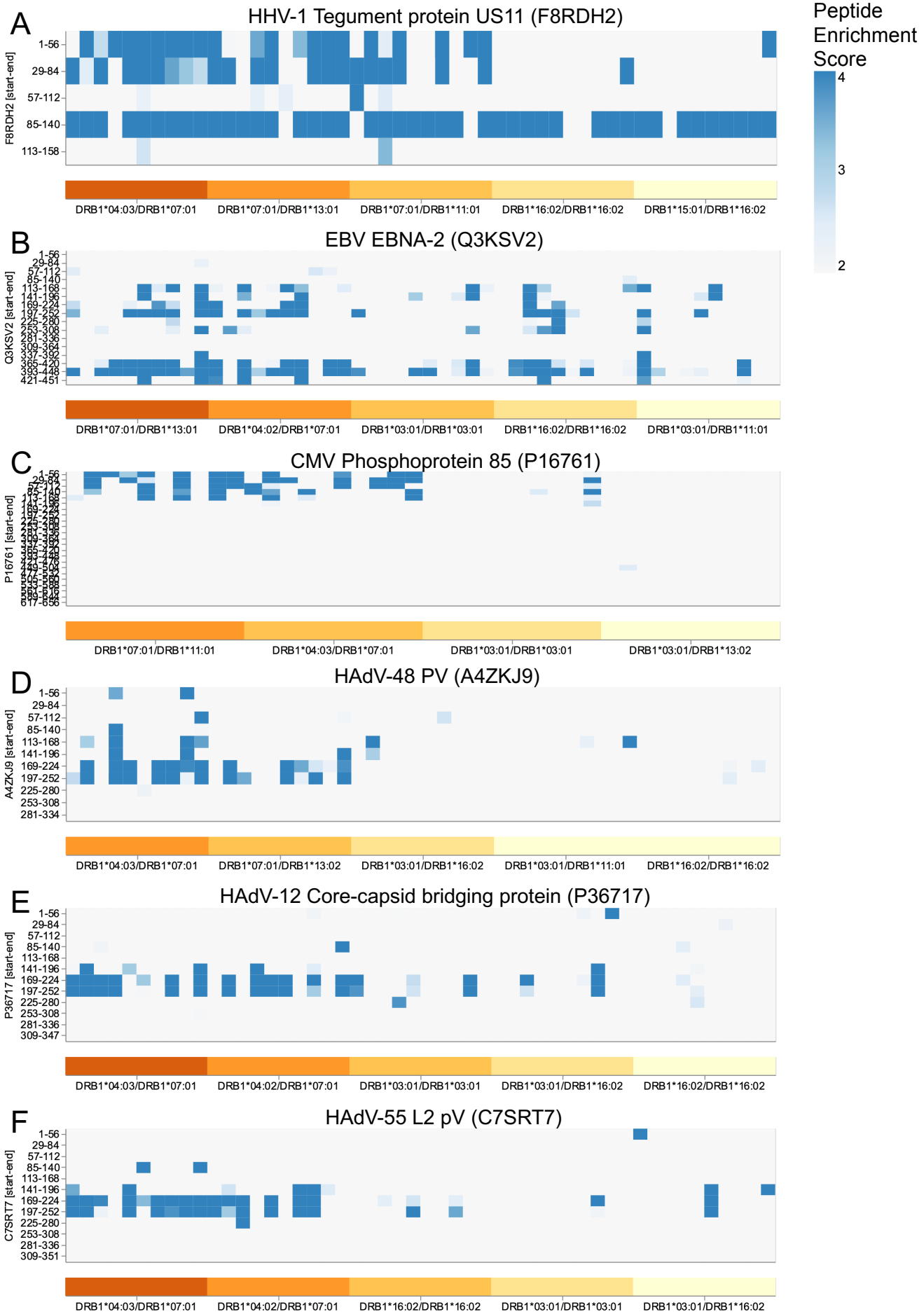

Supplement: Supplementary Table 1 — HLA genotypes for each study subject inferred by using HLA*LA and HLA-HD. [file DataSheet_1.zip › Supplementary Material/Supplementary Figure 5.PDF]

Supplementary Figure 6

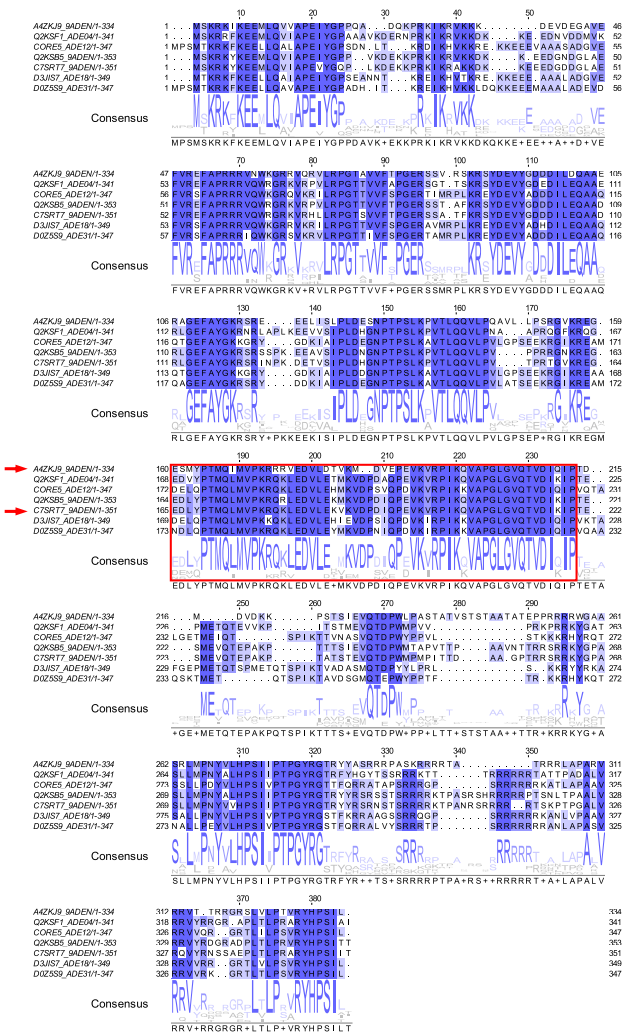

Supplement: Supplementary Table 1 — HLA genotypes for each study subject inferred by using HLA*LA and HLA-HD. [file DataSheet_1.zip › Supplementary Material/Supplementary Figure 6.PDF]
